# A phase IIb, randomised, parallel-group study: the efficacy, safety and tolerability of once-daily umeclidinium in patients with asthma receiving inhaled corticosteroids

**DOI:** 10.1186/s12931-020-01400-5

**Published:** 2020-06-12

**Authors:** Edward Kerwin, Steven Pascoe, Zelie Bailes, Robert Nathan, David Bernstein, Ronald Dahl, Robyn von Maltzahn, Kevin Robbins, Andrew Fowler, Laurie Lee

**Affiliations:** 1 Crisor LLC Research, Clinical Research Institute of Southern Oregon, Medford, OR USA; 2 GSK, Upper Providence, PA USA; 3 GSK, Stockley Park West, Uxbridge, Middlesex, UK; 4 Asthma & Allergy Associates, P.C. and Research Center, Colorado Springs, CO USA; 5 Division of Immunology, Allergy and Rheumatology, University of Cincinnati College of Medicine, Cincinnati, OH USA; 6 Bernstein Clinical Research Center, Cincinnati, OH USA; 7 GSK, 980 Great West Road, Brentford, Middlesex, UK

**Keywords:** Asthma, Forced expiratory volume in 1 s, Inhaled corticosteroid, Long-acting muscarinic antagonist, Umeclidinium

## Abstract

**Background:**

Patients with asthma uncontrolled on inhaled corticosteroids may benefit from umeclidinium (UMEC), a long-acting muscarinic antagonist.

**Methods:**

This Phase IIb, double-blind study included patients with reversible, uncontrolled/partially-controlled asthma for ≥6 months, receiving ≥100 mcg/day fluticasone propionate (or equivalent) for ≥12 weeks. Following a 2-week run-in on open-label fluticasone furoate (FF) 100 mcg, patients were randomised (1:1:1) to receive UMEC 31.25 mcg, UMEC 62.5 mcg or placebo on top of FF 100 mcg once-daily for 24 weeks. As-needed salbutamol was provided. Primary and secondary endpoints were change from baseline in clinic trough forced expiratory volume in 1 s (FEV_1_) and clinic FEV_1_ 3 h post-dose, respectively, at Week 24. Other endpoints included change from baseline in home daily spirometry (trough FEV_1_, evening FEV_1_, morning [pre-dose] and evening peak expiratory flow) over 24 weeks. Safety was assessed throughout the study.

**Results:**

The intent-to-treat population comprised 421 patients (UMEC 31.25 mcg: *n* =139, UMEC 62.5 mcg: *n* =139, placebo: *n* =143). UMEC 31.25 mcg and 62.5 mcg demonstrated significantly greater improvements from baseline in clinic trough FEV_1_ at Week 24 (difference [95% CI]: 0.176 L [0.092, 0.260; *p*<0.001] and 0.184 L [0.101, 0.268; *p*<0.001], respectively), clinic FEV_1_ 3 h post-dose at Week 24 (0.190 L [0.100, 0.279; *p*<0.001] and 0.198 L [0.109, 0.287; *p*<0.001], respectively) and mean change from baseline in daily home spirometry over 24 weeks versus placebo. No new safety signals were identified.

**Conclusions:**

UMEC is a highly effective bronchodilator that leads to improved lung function when administered as a single bronchodilator on top of FF in subjects with fully reversible, uncontrolled/partially-controlled moderate asthma. These data support a favourable benefit/risk profile for UMEC (31.25 mcg and 62.5 mcg).

**Trial registration:**

GSK study ID: 205832; Clinicaltrials.gov ID: NCT03012061.

## Background

Asthma, a common chronic condition, can occur at all ages [1–3]. In 2015, the prevalence of asthma was 358.2 million worldwide, reflecting an increase of 12.6% from 1990 [1]. Asthma is associated with respiratory symptoms and variable airflow limitation, and patients can be prone to exacerbations that may require treatment with systemic corticosteroids, urgent care visits or hospitalisations [2]. The Global Initiative for Asthma (GINA) 2019 strategy report recommends inhaled corticosteroids (ICS) as a controller treatment for patients with asthma [2]. ICS treatment can decrease inflammation, especially where there is evidence of a T-helper Type 2 allergic asthma phenotype [4]. Despite the availability of ICS treatment, which is often associated with improvements in symptom control, lung function and number of exacerbations [2], many patients remain uncontrolled; in 2010, 53.5% of treated patients with asthma in European countries were assessed as having not well-controlled asthma by the Asthma Control Test (trademark of QualityMetric Incorporated) [5].

Patients whose asthma symptoms remain uncontrolled with a history of exacerbations on ICS and a long-acting β_2_-agonist (LABA) may benefit from the addition of a long-acting muscarinic antagonist (LAMA) [2]. For example, tiotropium, a LAMA that has been approved as a long-term maintenance treatment for asthma in the United States (US) [6], the European Union [7] and Japan, has been associated with improvements in lung function and other clinical outcomes in patients with poorly controlled asthma despite receiving ICS or ICS/LABA therapy [8–10], with a similar magnitude of effect from adding LAMA to ICS as adding LABA to ICS [8, 11, 12].

The benefits of the LAMA umeclidinium (UMEC) on lung function are well established in chronic obstructive pulmonary disease (COPD) [13], and have also been described in patients with asthma and in those with features of both asthma and COPD [14, 15]. For example, improvements in trough forced expiratory volume in 1 s (FEV_1_) and pre-dose peak expiratory flow (PEF) have been reported for UMEC in combination with the ICS fluticasone furoate (FF) versus FF alone [14, 15]. To characterise the effects of UMEC in asthma and its longevity, this 24-week superiority study was conducted to evaluate the efficacy, safety and tolerability of once-daily UMEC 31.25 mcg or 62.5 mcg versus placebo in patients with asthma receiving FF 100 mcg who had previously been receiving ICS with or without a long-acting bronchodilator. UMEC 62.5 mcg was chosen as the upper dose based on findings from a previous study in which clinically meaningful improvements in lung function were reported versus FF alone [15]. The 31.25 mcg dose was selected for our study because the lowest UMEC dose used in the previous study (15.6 mcg) showed only modest improvements in lung function. Patients were required to have fully reversible asthma, with a post-bronchodilator FEV_1_/forced vital capacity (FVC) ≥0.7 [2], in order to exclude patients with fixed airway obstruction, in whom UMEC dose selection has previously been explored [15]. This study forms part of a clinical development programme for a closed triple ICS/LAMA/LABA therapy for asthma and will be used to help inform the choice of UMEC dose in the target population for FF/UMEC/VI. While the main focus of the study was to assess efficacy of UMEC on pulmonary function as measured by clinic and home spirometry, the Evaluating Respiratory Symptoms (E-RS) questionnaire [16, 17] was also included to evaluate its utility in patients with asthma with milder disease than have been assessed in previous studies [14, 15]. The E-RS is a respiratory symptom score developed and validated for use as part of the EXAcerbations of COPD (EXACT) instrument, a patient-reported outcome (PRO) measure for COPD, and subsequently evaluated in patients with features of both asthma and COPD [18].

## Materials and methods

### Study design

**Fig. 1:**
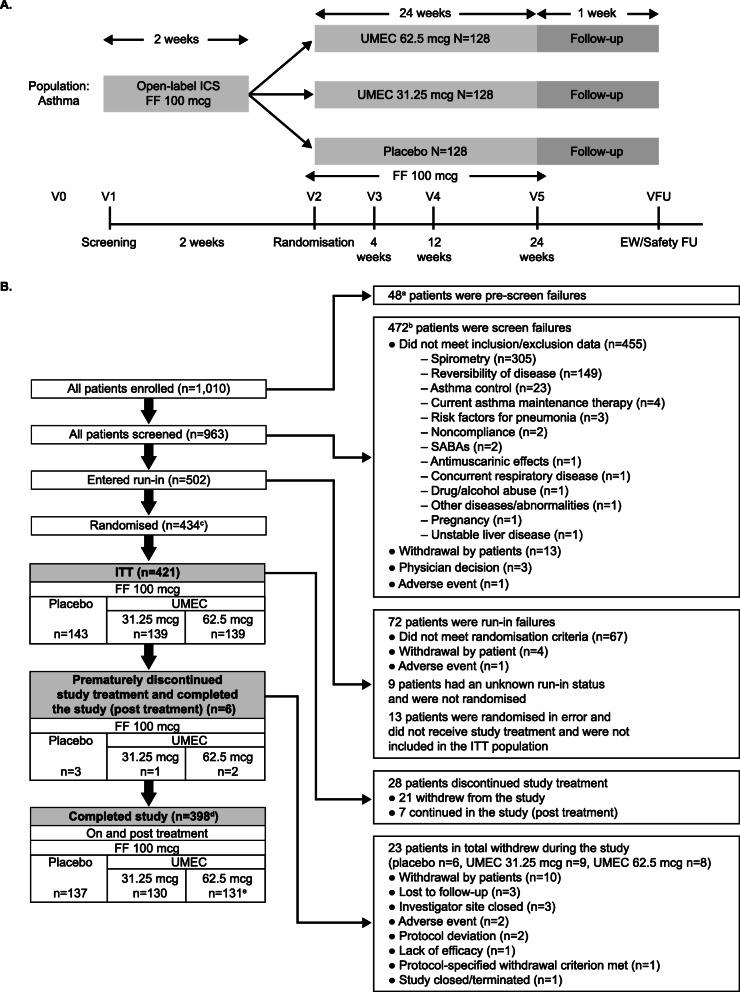
(**a**) Study design and (**b**) patient disposition. ^a^One patient failed pre-screening and entered screening, and was counted as both a pre-screen failure and in the all patients screened population. ^b^Eleven patients failed screening and entered the run-in period, and were counted as both screen failures and in the entered run-in population. ^c^The study planned to randomise 384 patients. ^d^One patient had an unknown study completion status. ^e^Patient 954 in the UMEC 62.5 mcg group discontinued study treatment the day prior to Visit 5 (Week 24) and was not dosed at that clinic visit. However, this patient was not reported as prematurely discontinuing study treatment in the eCRF and was counted in the completed study population. On treatment was defined as study treatment start date day (inclusive) to study treatment stop date + 1 (inclusive). Post treatment was defined as study treatment stop date + 1 day (exclusive) to Visit 5/EW Visit date (as applicable) (inclusive). The all patients enrolled population included all patients for whom a record exists in the database. The all patients screened population included all patients who completed ≥1 screening procedure. The randomised population included all patients who were randomised. The ITT population included all patients who were randomised, excluding those who were randomised in error and did not receive study treatment. AE, adverse event; eCRF, electronic Case Report Form; EW, early withdrawal; FF, fluticasone furoate; FU, follow-up; ICS, inhaled corticosteroid; ITT, intent-to-treat, SABA**,** short-acting β_2_-agonist; UMEC, umeclidinium; V, visit

This 24-week, Phase IIb, randomised, double-blind, placebo-controlled, parallel-group study (GSK study ID: 205832; Clinicaltrials.gov ID: NCT03012061) was conducted in Canada, Poland, Romania, Russian Federation and the US and compared the efficacy and safety of two doses of UMEC with placebo in patients with asthma. The study took place between January 2017 and May 2018. Following a pre-screening visit (Visit 0), asthma treatments were adjusted so that patients received ICS monotherapy without LABA or LAMA. At screening (Visit 1), patients entered a 2-week run-in period where their usual asthma therapy was stopped and replaced with open-label FF 100 mcg administered once daily via an ELLIPTA dry powder inhaler (DPI). Patients were then randomised (1:1:1) to receive UMEC 31.25 mcg, UMEC 62.5 mcg or placebo once daily (morning [AM]) for 24 weeks, administered via a separate ELLIPTA DPI, while remaining on FF 100 mcg (Fig. 1a). Following randomisation (Visit 2, Day 1), patients attended visits at Week 4 (Visit 3), Week 12 (Visit 4) and Week 24 (Visit 5), followed by a safety follow-up (FU) visit approximately 7 days after Visit 5. Patients who prematurely discontinued study treatment were encouraged to continue in the study for collection of post-treatment data, and patients who withdrew from the study at any time before completing a visit at Week 24 were encouraged to attend an early withdrawal visit. The study was divided into 4 study periods: screening/run-in (screening date to randomisation or treatment start date, if the first treatment dose was taken after the randomisation visit); on treatment (treatment start date to stop date plus 1 day); post treatment (treatment stop date plus 1 day to Visit 5/Early Withdrawal, visit inclusive for patients who continued in the study after discontinuing study treatment); and post study (any time after Visit 5/Early Withdrawal, all patients had a safety follow up 7 days after Visit 5/Early Withdrawal).

Salbutamol was provided to patients as a metered dose inhaler (MDI) for use during the study as a rescue medication. Salbutamol was withheld for ≥6 h prior to Visits 2–5/Early Withdrawal Visit (as applicable) and home spirometry was to be performed prior to any salbutamol use. Additional asthma medications for exacerbations were permitted at the discretion of the investigator. A summary of permitted and prohibited medications is included in Supplementary Table 1.

Patients were assigned to treatment using a randomisation schedule generated with a validated computerised system using RAMOS NG software. Double-blinding was ensured by administering study drug and placebo using matched ELLIPTA DPIs.

### Patient sample

#### Inclusion and exclusion criteria

At Visit 0 (pre-screening), patients were required to be ≥18 years of age with a diagnosis of asthma (as defined by National Institutes of Health [3]) for ≥6 months, receiving continuous therapy with ICS ≥100 mcg/day fluticasone propionate (or equivalent) ± LABA or LAMA for ≥12 weeks with no change in asthma therapy for the previous 4 weeks and able to withhold rescue medication for ≥6 h before each clinic visit.

At screening, patients were required to have an Asthma Control Questionnaire (ACQ)-6 score >0.75, and therefore considered to have either partially controlled (ACQ-6 score >0.75 and <1.5) or uncontrolled asthma (ACQ-6 score ≥1.5). Patients were also required to have a pre-bronchodilator AM FEV_1_ ≤90% predicted, AND a post-bronchodilator FEV_1_/FVC ≥0.7 and evidence of reversibility to salbutamol (≥12% and ≥200 mL increase in FEV_1_ 20–60 min following inhalation), referred to here as fully reversible asthma.

Patients who had x-ray-documented pneumonia, a severe asthma exacerbation within 12 weeks prior to screening, pneumonia risk factors such as immune suppression or neurological disorders affecting control of the upper airway at screening were excluded, as were those who had evidence of a concurrent respiratory disease (including emphysema or COPD) or current and clinically significant disease of the major body systems and uncontrolled haematological abnormalities. Those with current unstable liver disease, clinically significant electrocardiogram abnormalities, current unstable and life-threatening cardiac disease, conditions that may be affected by antimuscarinic use, or cancer within the previous 5 years were also excluded. Current and former smokers with a history of ≥10 pack years and inhaled tobacco use in the previous 12 months were also excluded, in order to omit patients who may have a COPD component.

#### Randomisation criteria

Patients were eligible for randomisation if they continued to have an ACQ-6 score >0.75 and an AM pre-bronchodilator FEV_1_ ≤90% predicted at Visit 2, were compliant with the electronic diary (eDiary) on ≥4/7 final days of the run-in period and had no change in asthma therapy during the run-in period. Patients who experienced a moderate/severe asthma exacerbation or a respiratory infection during the run-in period were not eligible to be randomised.

### Endpoints

The primary efficacy endpoint was mean change from baseline (CFB) in clinic trough FEV_1_ at Week 24. The secondary efficacy endpoint was mean CFB in clinic FEV_1_ 3 h post dose at Week 24. Other efficacy endpoints included mean CFB in home spirometry readings (trough FEV_1_, evening [PM] FEV_1_, AM PEF and PM PEF) and mean rescue medication use (puffs/day) over the 24-week period, mean CFB in PROs (St George’s Respiratory Questionnaire [SGRQ] total and domain scores, Asthma Quality of Life Questionnaire [AQLQ] total score, ACQ-5 total score and E-RS total score) and percentage of patients meeting a responder threshold for CFB in SGRQ total score (≥4 points improvement [decrease]) [19], AQLQ total score (≥0.5 points improvement [increase]) [20] and ACQ-5 total score (≥0.5 points improvement [decrease]) [21], annualised rate of moderate/severe and severe asthma exacerbations and time to first moderate/severe and severe exacerbation. Safety endpoints included incidence of adverse events (AEs), serious AEs (SAEs) and AEs of special interest (AESIs) as well as electrocardiogram measurements and vital signs throughout the study.

### Assessments

FEV_1_ (pre- and post-dose) and SGRQ, AQLQ and ACQ-5 scores were assessed during clinic visits. An eDiary was used to record AM (pre-study treatment or pre-run-in treatment, as applicable) and PM (pre-rescue bronchodilator) PEF. In the morning, PEF was captured upon waking at 6–11 AM. Home trough and PM FEV_1_, rescue medication use and E-RS total score were also recorded using the eDiary. The E-RS total score consisted of the 11 respiratory symptom items from the 14-item EXACT: COPD PRO measure (score: 0–40, minimal clinically important difference [MCID]: ≥− 2 points) [16, 17]. Night-time wakening, asthma symptoms and physical activity were also recorded, though not analysed as endpoints. Exacerbations, emergency department visits and hospitalisations were reported by patients using a medical problems/medications taken worksheet. A moderate exacerbation was defined as a deterioration in asthma symptoms, a deterioration in lung function or increased rescue bronchodilator use lasting ≥2 days that did not require ≥3 days of systemic corticosteroid use and/or hospitalisation [22, 23]. A severe exacerbation was defined as a deterioration of asthma that required either systemic corticosteroids for ≥3 days or hospitalisation or an emergency department visit due to asthma that required systemic corticosteroids [22]. Exacerbations that occurred within 7 days of the previous exacerbation were treated as a continuation of the same exacerbation.

### Statistical analyses

With a sample size of 115 patients per treatment group, the study had 80% power to detect a statistically significant difference in trough FEV_1_ at Week 24 at the 2-sided 5% level, assuming a true treatment difference of 130 mL and standard deviation (SD) of 350 mL [8, 15]. Randomisation of 128 patients per treatment group was planned to allow 10% missing data. The intent-to-treat (ITT) population, which included all patients randomised except those randomised in error and not treated, was used for the efficacy and safety analyses.

This was a superiority study designed to demonstrate the benefit of UMEC 31.25 and 62.5 mcg versus placebo in patients on background therapy of FF 100 mcg. Multiplicity across the primary treatment comparisons for the primary endpoint was controlled using a step-down procedure based on a statistical hierarchy, firstly testing UMEC 62.5 mcg versus placebo, and then testing UMEC 31.25 mcg versus placebo. For the secondary and other endpoints, both UMEC doses versus placebo were also compared without adjustment for multiplicity. In addition, UMEC 31.25 mcg was compared with UMEC 62.5 mcg in an exploratory manner for all analyses. No *p*-values are reported for these endpoints and we instead include treatment estimates and 95% confidence intervals (CI).

The primary efficacy endpoint was analysed using a mixed model repeated measures (MMRM) that included covariates of treatment group, sex, region, visit, age, and baseline value, and interactions of baseline value by visit and treatment group by visit. The secondary endpoint was evaluated using an analysis of covariance adjusting for covariates as per the primary efficacy analysis above, excluding visit and the by visit interaction terms. Home spirometry, rescue medication use and E-RS total score were analysed by deriving mean values over 4-weekly incremental periods from Week 1 to Week 24. Additionally, mean weekly values over Weeks 1–8 were derived for trough FEV_1_. A minimum of 50% of data was required for a period to derive an analysis value. An MMRM model adjusted for the covariates treatment, age, sex, region, baseline value, period, treatment by period interaction and baseline value by period interaction was used. An estimate over Weeks 1–24 was obtained for these endpoints from the MMRM analysis. SGRQ total and domain scores and total scores for AQLQ and ACQ-5 were analysed using a MMRM model similar to that used for the primary endpoint but adapted for the endpoint being analysed. Responder analyses for the PRO measures were conducted using a generalised linear model for binary outcome measures, appropriately adjusted for covariates as above. Exacerbation rates were analysed using a generalised linear model assuming a negative binomial distribution with log (time on study) as an offset variable, and time to first exacerbation was analysed using a Cox’s proportional hazards model. Both models were appropriately adjusted for covariates as above. Selected safety endpoints (systolic blood pressure, diastolic blood pressure, pulse rate, QT interval corrected for pulse rate by Fridericia’s formula, PR interval) were analysed using a similar MMRM model to the primary efficacy analysis, adapted for the endpoint being analysed.

A tipping point and a jump to reference sensitivity analysis were performed on mean CFB in clinic trough FEV_1_ at Week 24, based on the ITT population. These analyses explored the impact of missing data by multiply imputing the unobserved data based on different assumptions in each treatment group. For each, imputation considered the same covariates in the model as the primary efficacy analysis modelled at each visit. The tipping point analysis assumed a range of scenarios for participants who withdrew, which varied independently for the two UMEC treatment groups and the placebo group. Results were used to explore the conditions under which the significant difference between UMEC 62.5 mcg versus placebo and UMEC 31.25 mcg versus placebo no longer held true. The jump to reference analysis assumed patients in the UMEC groups who withdrew from the study would have provided data similar to that observed in the placebo group. The SAS 9.4 package was used for all statistical analyses.

For eDiary endpoints (home spirometry, rescue medication use, E-RS total score), the baseline value was the final 14 days of the run-in period prior to initiating study treatment. A minimum of 7 days data was required to derive a baseline value. Baseline for PRO scores was the score determined at randomisation (Visit 1). For clinic FEV_1_, the baseline measurement was the last measurement prior to the start of randomised treatment.

## Results

### Patients

Of the 1,010 patients enrolled, 963 were screened, 434 were randomised and 398 completed the study. The ITT population included 421 patients (UMEC 31.25 mcg: *n* =139, UMEC 62.5 mcg: *n* =139, placebo: *n* =143) as 13 patients were randomised in error and were not treated. Of those screened, 472 (47% of the enrolled population) patients failed screening, mainly due to failure to meet the inclusion/exclusion criteria (*n* =455 [96%]), particularly the spirometry and/or reversibility criteria (Fig. 1b). Of the enrolled patients, 7% were considered run-in failures, mainly due to not meeting the randomisation criteria (93%) of compliance with eDiary reporting (35%) and not meeting the FEV_1_ % predicted criterion (33%).

Patients had a mean (SD) age of 48.8 (14.6) years, most (71%) were female, had never smoked (92%), and were inadequately controlled as shown by PRO scores (Table 1). The mean (SD) age of asthma onset and duration was 33.8 (17.23) and 14.8 (13.63) years, respectively (Table 1). The study population had inadequately controlled asthma at baseline, with mean (SD) total scores for ACQ-6 and SGRQ of 1.7 (0.5) and 36 (17), respectively, and a mean (SD) baseline use of rescue salbutamol of 1.2 (1.4) puffs per day. Mean (SD) reversibility to salbutamol was 28.3% (19.4) and patients displayed moderate pre-bronchodilator airflow obstruction with mean (SD) FEV_1_ % predicted of 68.6% (11.7). Fifteen percent of patients experienced one or more severe asthma exacerbations in the prior year (Table 1). All baseline demographic parameters were similar across treatment groups. Minor differences in clinical characteristics (Table 1) and regional demographics were noted that were not considered to influence the study conclusions (Supplementary Table 2).

**Table 1:** Baseline demographics and clinical characteristics at screening and randomisation (ITT population)

	Placebo (*N* =143)	UMEC 31.25 mcg (*N* =139)	UMEC 62.5 mcg (*N* =139)	Total (*N* =421)
Age*, years	49.3 (13.93)	48.7 (15.83)	48.5 (14.21)	48.8 (14.64)
**Female, n (%)**	106 (74)	94 (68)	98 (71)	298 (71)
**Race: White, n (%)**	131 (92)	127 (91)	129 (93)	387 (92)
**BMI, kg/m**^**2**^	29.89 (7.31)	29.40 (7.52)	29.02 (7.68)	29.44 (7.49)
**Smoking status, n (%)**				
**Never smoked**	127 (89)	132 (95)	129 (93)	388 (92)
**Former smoker**	16 (11)	7 (5)	10 (7)	33 (8)
**Number of pack years, mean (SD)**	4.05 (2.74)	4.14 (2.70)	1.85 (1.79)	3.40 (2.62)
**Duration of asthma, years**	15.06 (14.40)	13.82 (12.92)	15.61 (13.54)	14.83 (13.63)
**Age of onset of asthma**^**†**^**, years**	33.9 (16.71)	34.7 (17.90)	32.6 (17.13)	33.8 (17.23)
**Pre-bronchodilator FEV**_**1**_**, L**	*n* =143	*n* =137	*n* =139	*n* =419
	2.11 (0.71)	2.18 (0.72)	2.15 (0.60)	2.14 (0.68)
**Pre-bronchodilator predicted FEV**_**1**_**, %**	*n* =143	*n* =137	*n* =139	*n* =419
	67.90 (12.09)	68.85 (12.00)	69.11 (11.12)	68.61 (11.72)
**Pre-bronchodilator FEV**_**1**_**/FVC, ratio**	*n* =143	*n* =137	*n* =139	*n* =419
	0.70 (0.08)	0.70 (0.09)	0.72 (0.09)	0.71 (0.09)
**Reversibility, %**	*n* =142	*n* =137	*n* =138	*n* =417
	25.97 (15.89)	28.21 (18.64)	30.75 (22.84)	28.29 (19.36)
**SGRQ total score**^¶^	36.96 (17.45)	35.56 (15.75)	36.30 (18.06)	36.28 (17.09)
**AQLQ total score**	5.03 (0.93)	5.10 (0.87)	5.14 (0.80)	5.09 (0.87)
**ACQ-6 score**^¶^	1.70 (0.55)	1.69 (0.54)	1.59 (0.53)	1.66 (0.54)
**ACQ-6 control category**^¶§^**, n (%)**				
**Partially controlled**	51 (36)	53 (38)	60 (43)	164 (39)
**Inadequately controlled**	92 (64)	85 (62)	79 (57)	256 (61)
**E-RS total score**^±^	7.58 (5.58)	7.20 (5.11)	7.44 (5.52)	7.41 (5.40)
**Rescue medication use**^±^**, puffs/day**	1.30 (1.46)	1.30 (1.61)	1.00 (1.21)	1.20 (1.44)
**Number of severe exacerbations in the 12 months prior to screening, n (%)**				
**0**	123 (86)	108 (78)	128 (92)	359 (85)
**1**	16 (11)	22 (16)	9 (6)	47 (11)
**≥2**	4 (3)	9 (6)	2 (1)	15 (4)

Data are mean (SD) unless otherwise stated

*Age is derived using the date of the pre-screening visit. Only year of birth is collected. Day and month of birth are imputed as 30 June. ^**†**^Calculated based on age and duration of asthma at pre-screening. ^¶^Baseline value was defined as score recorded at the Randomisation visit (Visit 2/Day 1).

^§^ACQ-6 score 0.75 to <1.5: partially controlled; ACQ-6 score ≥1.5: inadequately controlled. ^±^Baseline value was defined as the mean over the last 14 days during the run-in period prior to study treatment start date (Randomisation).

*ACQ-6* Asthma Control Questionnaire-6, *AQLQ* Asthma Quality of Life Questionnaire, *BMI* body mass index, *E-RS total score* Evaluating Respiratory Symptoms, *FEV*_*1*_ forced expiratory volume in 1 s, *FVC* forced vital capacity, *ITT* intent-to-treat, *SD* standard deviation, *SGRQ* St George’s Respiratory Questionnaire, *UMEC* umeclidinium

### Clinic FEV_1_

**Fig. 2:**
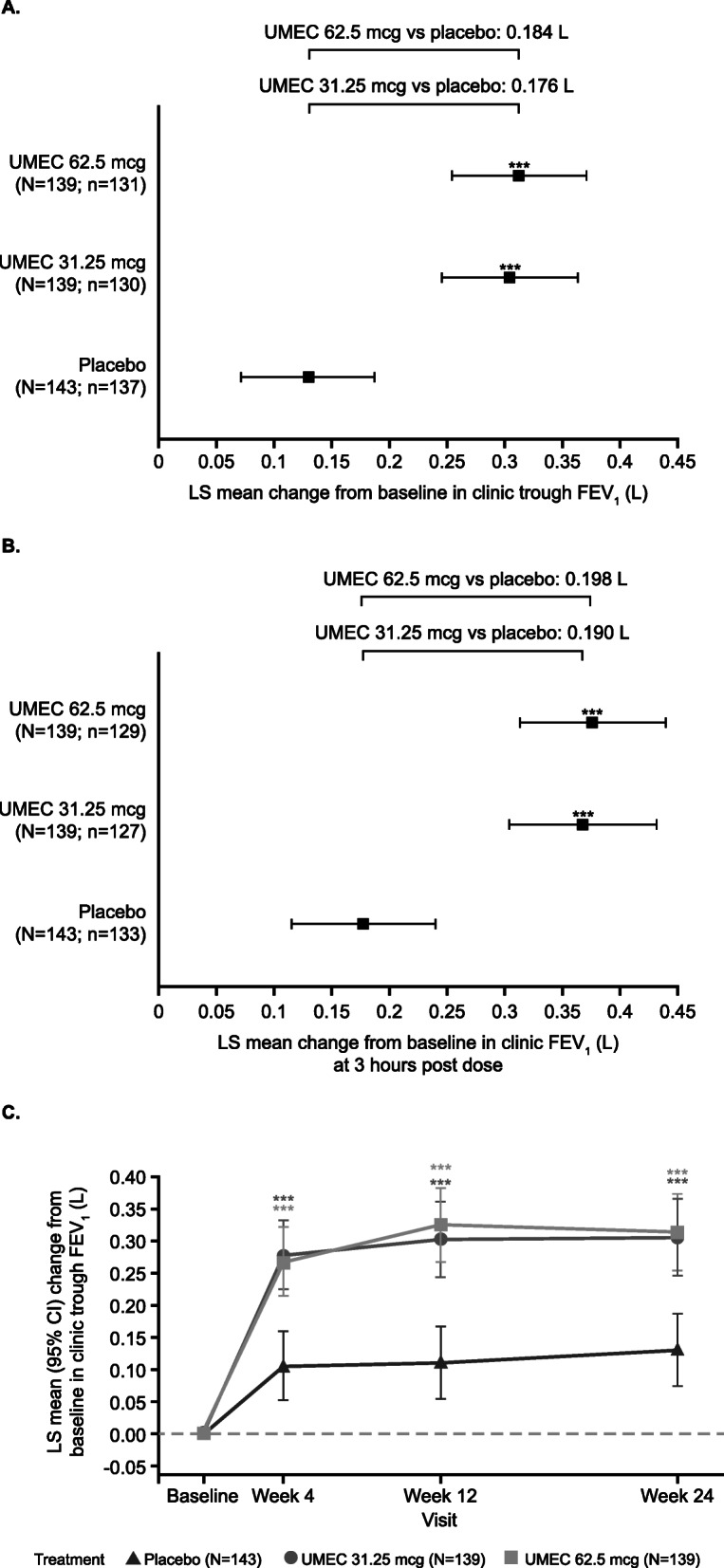
LS mean change from baseline in clinic spirometry measures (ITT population). LS mean (95% CI) change from baseline in clinic (**a**) trough FEV_1_ at Week 24, (**b**) FEV_1_ (L) at 3 h post dose at Week 24 and (**c**) trough FEV_1_ at Weeks 4, 12 and 24 (ITT population). Error bars represent 95% CI. ***p<0.001, treatment difference from placebo. CI, confidence interval; FEV_1_, forced expiratory volume in 1 s; ITT, intent-to-treat; LS, least squares; UMEC, umeclidinium; N, ITT population; n, number of participants with analysable data at Week 24

At Week 24, all treatments administered with FF, including placebo, were associated with increases in the least squares (LS) mean (95% CI) CFB in clinic trough FEV_1_ (placebo: 0.129 L [0.070, 0.187], UMEC 31.25 mcg: 0.305 L [0.245, 0.364], UMEC 62.5 mcg: 0.313 L [0.254, 0.372]; Fig. 2a) and clinic FEV_1_ 3 h post dose (placebo: 0.177 L [0.114, 0.239], UMEC 31.25 mcg: 0.366 L [0.302, 0.430], UMEC 62.5 mcg: 0.374 L [0.311, 0.438]; Fig. 2b). For trough FEV_1_, the increase versus placebo was significantly greater for both UMEC doses at Week 24, with a difference (95% CI) of 0.176 L (0.092, 0.260; *p*<0.001) for UMEC 31.25 mcg and 0.184 L (0.101, 0.268; *p*<0.001) for UMEC 62.5 mcg (Fig. 2a). For both doses of UMEC versus placebo, the sensitivity analyses demonstrated that the primary efficacy outcome was robust. These findings were supported by the CFB in clinic FEV_1_ 3 h post dose at Week 24, which was significantly greater with UMEC 31.25 mcg and 62.5 mcg compared with placebo, with differences (95% CI) of 0.190 L (0.100, 0.279; *p*<0.001) and 0.198 L (0.109, 0.287; *p*<0.001), respectively (Fig. 2b). CFB in clinic trough FEV_1_ was also significantly greater at Weeks 4 and 12 for both UMEC 31.25 mcg and 62.5 mcg versus placebo (Fig. 2c).

### Other efficacy endpoints

Over the 24-week treatment period (Weeks 1–24), increases in LS mean CFB in home trough FEV_1_ were observed with UMEC 31.25 mcg (0.036 L, 95% CI: − 0.012, 0.084) and UMEC 62.5 mcg (0.061 L, 95% CI: 0.013, 0.109) but not with placebo, which was associated with a small FEV_1_ reduction (− 0.038 L, 95% CI: − 0.085, 0.010). Treatment differences versus placebo were 0.074 L (0.006, 0.141; *p*=0.033) for UMEC 31.25 mcg and 0.098 L (0.031, 0.166; *p*=0.004) for UMEC 62.5 mcg (Table 2).

**Table 2:** Effect of UMEC 31.25 and 62.5 mcg versus placebo on home trough FEV_1_

	Time point	Placebo (*N* =143)	UMEC 31.25 mcg (*N* =139)	UMEC 62.5 mcg (*N* =139)
n	Weeks −2 and − 1 (BL)	143	138	139
mean (SD)		2.222 (0.805)	2.304 (0.718)	2.248 (0.662)
n	Weeks 1–4	141	135	138
LS mean change (SE)		−0.034 (0.023)	0.029 (0.023)	0.046 (0.023)
Difference vs placebo (95% CI)			0.064 (0.000, 0.127)	0.080 (0.017, 0.144)
n	Weeks 5–8	139	128	135
LS mean change (SE)		− 0.046 (0.027)	0.018 (0.027)	0.063 (0.027)
Difference vs placebo (95% CI)			0.064 (−0.011, 0.139)	0.109 (0.034, 0.184)
n	Weeks 9–12	135	130	132
LS mean change (SE)		−0.058 (0.027)	0.045 (0.027)	0.054 (0.027)
Difference vs placebo (95% CI)			0.103 (0.028, 0.178)	0.112 (0.037, 0.186)
n	Weeks 13–16	128	124	125
LS mean change (SE)		−0.026 (0.028)	0.052 (0.028)	0.058 (0.028)
Difference vs placebo (95% CI)			0.079 (0.000, 0.157)	0.084 (0.006, 0.162)
n	Weeks 17–20	126	123	124
LS mean change (SE)		−0.023 (0.029)	0.039 (0.029)	0.065 (0.029)
Difference vs placebo (95% CI)			0.061 (−0.019, 0.142)	0.087 (0.007, 0.167)
n	Weeks 21–24	128	123	125
LS mean change (SE)		−0.038 (0.029)	0.033 (0.030)	0.081 (0.029)
Difference vs placebo (95% CI)			0.071 (−0.011, 0.152)	0.118 (0.037, 0.199)
n	Weeks 1–24*	142	137	138
LS mean change (SE)		−0.038 (0.024)	0.036 (0.025)	0.061 (0.024)
Difference vs placebo (95% CI)			0.074 (0.006, 0.141)	0.098 (0.031, 0.166)

*Treatment effect averaged over all time points. These analyses were performed using an MMRM model with covariates of treatment, age, sex, region, baseline value and 4-weekly period, and with interaction terms for baseline by period and treatment by period.

*BL* baseline, *CI* confidence interval, *FEV*_*1*_ forced expiratory volume in 1 s, *LS* least squares, *MMRM* mixed model repeated measures, *SD* standard deviation, *SE* standard error, *UMEC* umeclidinium

**Fig. 3:**
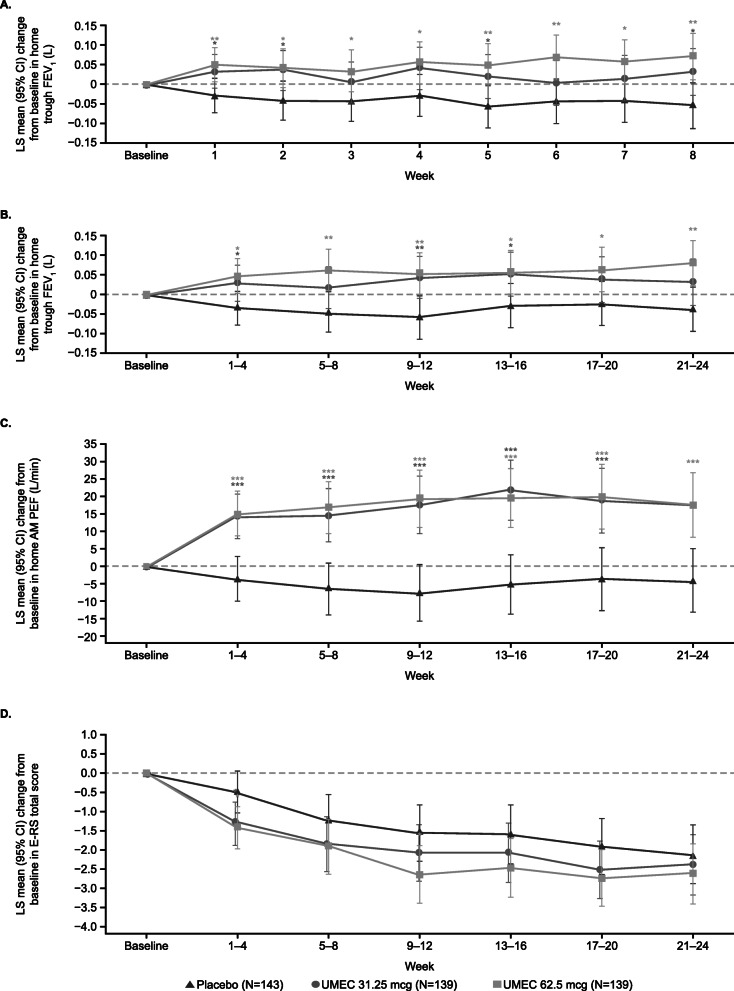
LS mean change from baseline in home spirometry measures and E-RS total scores. LS mean (95% CI) change from baseline in (**a**) home trough FEV_1_ up to Week 8 by 1-weekly intervals, (**b**) home trough FEV_1_ over the 24-week treatment period by 4-weekly intervals, (**c**) AM PEF over the 24-week treatment period by 4-weekly intervals and (**d**) E-RS total scores over the 24-week treatment period by 4-weekly intervals (ITT population). Error bars represent 95% CI. *p≤0.05; **p≤0.01; ***p≤0.001, treatment difference from placebo. AM, morning; CI, confidence interval; E-RS, Evaluating Respiratory Symptoms; FEV_1_, forced expiratory volume in 1 s; ITT, intent-to-treat; LS, least squares, PEF, peak expiratory flow; UMEC, umeclidinium

When home trough FEV_1_ measurements were considered over weekly intervals during Weeks 1–8, there were significant increases from Week 1 with both doses of UMEC versus placebo (Fig. 3a).

Over 24 weeks, considering weekly intervals, UMEC 62.5 mcg showed statistically significant improvements versus placebo for home trough FEV_1_ during each period. UMEC 31.25 mcg also showed improvements versus placebo over the 24-week period; however, they were smaller than for UMEC 62.5 mcg and only met statistical significance (*p*<0.05) for three of the six periods. Placebo showed a decline up until Week 12 (Table 2, Fig. 3b). Greater CFB in home trough FEV_1_ over the Week 21–24 period was observed with both UMEC doses versus placebo, with treatment differences (95% CI) of 0.071 L (− 0.011, 0.152; *p*=0.089) for UMEC 31.25 mcg and 0.118 (0.037, 0.199; *p*=0.004) for UMEC 62.5 mcg (Table 2).

Significantly greater improvements from baseline in home PM FEV_1_, AM pre-dose PEF and PM PEF with both doses of UMEC versus placebo were also seen over the 24-week treatment period, with a decline from the baseline in placebo for all three measures (Weeks 1–24; Supplementary Table 3). For AM PEF, significant improvements with both UMEC doses versus placebo occurred early (by Weeks 1–4) and were maintained over the treatment period (Fig. 3c), with similar results for PM PEF (data not shown).

At Week 24, reduced rescue medication use was seen in all treatment groups, with no significant differences between either of the UMEC doses and placebo (Supplementary Table 4).

### PRO endpoints

For all PRO measures (SGRQ total and domain scores, AQLQ total score, ACQ-5 total score and E-RS total score), improvements from baseline were seen in all three treatment groups at Week 24. The changes were greater than the MCIDs for SGRQ total and domain scores, AQLQ total score and ACQ-5 total score [19–21, 24]; the E-RS does not have an accepted MCID in asthma, nevertheless it was reduced by at least 2 units in both UMEC groups (Supplementary Table 4). With the exception of SGRQ impact score for UMEC 31.25 mcg and AQLQ total score for UMEC 62.5 mcg, patients treated with either dose of UMEC consistently showed numerically greater improvements in PRO scores at Week 24 compared with those who were treated with placebo, although differences were not significant (Supplementary Table 4).

For E-RS total score, statistically significant improvements from baseline with UMEC 31.25 mcg and UMEC 62.5 mcg versus placebo were seen at Weeks 1–4; however, while numerical differences were observed between the UMEC groups and placebo during the rest of the treatment period, these differences were not statistically significant (Fig. 3d).

Overall, over half of patients met the responder thresholds for SGRQ, AQLQ and ACQ-5 total scores across the treatment groups (Supplementary Table 5). For SGRQ, AQLQ and ACQ-5 total scores, there were no statistically significant differences between the odds of being a responder for UMEC 31.25 mcg or 62.5 mcg versus placebo.

### Exacerbations

Few patients experienced a moderate/severe exacerbation during the study across treatment groups (placebo: 15%; UMEC 31.25 mcg: 11%; UMEC 62.5 mcg: 12%). An equal number of patients experienced a moderate exacerbation (7%) in each treatment group; however, more patients experienced a severe exacerbation in the placebo group (8%) than either UMEC group (4%). A higher number of severe exacerbations were reported in the placebo group (16 events) compared with the UMEC 31.25 mcg (6 events) and 62.5 mcg (7 events) groups. The annualised rates of moderate/severe and severe exacerbations were numerically lower with either dose of UMEC compared with placebo; however, this was only statistically significant for severe exacerbations with UMEC 31.25 mcg versus placebo (rate ratio [95% CI]: 0.33 [0.12, 0.90]; *p*=0.030; Supplementary Table 6).

### Safety

The incidence of on-treatment AEs was higher in the UMEC 31.25 mcg group (53%) compared with the placebo (45%) and UMEC 62.5 mcg (41%) groups (Table 3). This difference did not appear to be driven by any single system organ class or preferred term. Common on-treatment AEs included nasopharyngitis, headache and upper respiratory tract infection (Table 3). The incidences of drug-related AEs and SAEs were low and similar across the treatment groups (Table 3). There were no fatalities. The most common AESIs included dry mouth/drying of the airway secretions, local steroid effects and cardiovascular effects (particularly hypertension), and their occurrences were similar between treatment groups (Table 4). There was a statistically significant LS mean increase from baseline in pulse rate for UMEC 62.5 mcg compared with placebo at Weeks 12 and 24 (Week 12: 1.7 beats/min [95% CI: 0.00, 3.30], *p*=0.045; Week 24: 3.4 beats/min [95% CI: 1.30, 5.50], *p*=0.002). This observation was at least partially driven by a reduction in the placebo group of 0.6 and 1.6 beats/min respectively for Weeks 12 and 24, and not observed on electrocardiogram. No clinically relevant findings were observed for electrocardiogram or vital signs.

**Table 3:** On-treatment AEs occurring in ≥3% of patients in any treatment group (ITT population)

	Placebo (*N* =143) *n* (%)	UMEC 31.25 mcg (*N* =139) *n* (%)	UMEC 62.5 mcg (*N* =139) *n* (%)
Any AE	65 (45)	73 (53)	57 (41)
Nasopharyngitis	17 (12)	14 (10)	13 (9)
Upper respiratory tract infection	3 (2)	8 (6)	6 (4)
Respiratory tract infection viral	5 (3)	7 (5)	4 (3)
Oropharyngeal pain	2 (1)	6 (4)	4 (3)
Dysphonia	2 (1)	6 (4)	0
Headache	11 (8)	9 (6)	12 (9)
Toothache	4 (3)	1 (<1)	5 (4)
Back pain	5 (3)	3 (2)	1 (<1)
Arthralgia	1 (<1)	5 (4)	2 (1)
Hypertension	5 (3)	4 (3)	1 (<1)
Drug-related AEs	4 (3)	6 (4)	3 (2)
AEs leading to permanent discontinuation of study treatment	1 (<1)	1 (<1)	1 (<1)
AEs leading to withdrawal from the study	1 (<1)	0	0
Any SAE	5 (3)	4 (3)	3 (2)
Drug-related SAEs	0	0	0
Fatal SAEs	0	0	0
Fatal drug-related SAEs	0	0	0

AEs listed by preferred term

*AE* adverse event, *ITT* intent-to-treat, *SAE* serious AE, *UMEC* umeclidinium

**Table 4:** Summary of on-treatment AESIs (ITT population)

	Placebo (*N* =143) *n* (%)	UMEC 31.25 mcg (*N* =139) *n* (%)	UMEC 62.5 mcg (*N* =139) *n* (%)
Adrenal suppression	0	0	0
Anticholinergic syndrome (SMQ)*	1 (<1)	1 (<1)	3 (2)
CV effects*	10 (7)	7 (5)	6 (4)
Hypertension (SMQ)	7 (5)	5 (4)	4 (3)
Cardiac arrhythmia	1 (<1)	1 (<1)	1 (<1)
Ischaemic heart disease (SMQ)	1 (<1)	0	1 (<1)
Cardiac failure (SMQ)	1 (<1)	1 (<1)	0
CNS haemorrhages and cerebrovascular conditions (SMQ)	1 (<1)	0	0
Decreased BMD and associated fractures	0	0	0
Dry mouth/drying of the airway secretions (broad focus)*	28 (20)	29 (21)	27 (19)
Dry mouth/drying of the airway secretions (narrow focus)*	0	0	1(<1)
Gastrointestinal obstruction (SMQ)*	0	0	0
Hyperglycaemia/ new onset diabetes mellitus (SMQ)	1 (<1)	0	0
Hypersensitivity*	3 (2)	3 (2)	1 (<1)
Infective pneumonia (SMQ)*	1 (<1)	1 (<1)	0
LRTI (excluding infective pneumonia SMQ)*	3 (2)	2 (1)	6 (4)
Local steroid effects	5 (3)	12 (9)	6 (4)
Ocular effects*	0	1 (<1)	0
Glaucoma (SMQ)	0	1 (<1)	0
Lens disorders (SMQ)	0	0	0
Urinary retention*	1 (<1)	0	0

*Special interest groups related to UMEC and LAMAs

*AESI* adverse event of special interest, *BMD* bone mineral density, *CNS* central nervous system, *CV* cardiovascular, *ITT* intent-to-treat, *LAMA* long-acting muscarinic antagonist, *LRTI* lower respiratory tract infections, *MedDRA* Medical Dictionary for Regulatory Activities, *SMQ* standardised MedDRA queries, *UMEC* umeclidinium

## Discussion

This is the first study to examine the effect of UMEC in patients with asthma and fully reversible airflow obstruction over a 24-week period. Results show that UMEC is an effective bronchodilator in this particular asthma population receiving FF as background therapy. Specifically, UMEC 31.25 and 62.5 mcg demonstrated statistically significant and clinically meaningful improvements in the primary endpoint of clinic trough FEV_1_ at Week 24 compared with placebo. While an MCID in FEV_1_ has not been formally defined in asthma, a previous Phase III study of patients with persistent asthma showed that FF 100 mcg and FF/VI 100/25 mcg improved trough FEV_1_ by 136 mL and 172 mL, respectively, compared with placebo [25]. We report changes from baseline versus placebo in clinic trough FEV_1_ of 176 mL for UMEC 31.25 mcg and 184 mL for UMEC 62.5 mcg at Week 24; these changes are greater than the change reported with FF alone and equal to that achieved with FF/VI combined. In addition, in COPD it has been widely reported that a change in FEV_1_ of 100 mL is perceptible by patients and correlates with fewer relapses [26].

These results were supported by significantly greater CFB with both UMEC doses versus placebo in the secondary endpoint of clinic FEV_1_ 3 h post dose at Week 24, as well as in other endpoints of daily home trough FEV_1_ and PEF over Weeks 1–24. The improvements with UMEC in clinic trough and 3 h post dose FEV_1_ are of similar magnitude to that of tiotropium in patients with asthma uncontrolled on ICS [8]. Additionally, improvements in FEV_1_ and PEF in the current study are in line with those seen in patients with asthma treated with vilanterol in addition to FF [12].

Home spirometry has been used in other clinical studies of respiratory conditions including post lung transplantation, idiopathic pulmonary fibrosis, COPD and asthma [27–30]. In the current study, the daily monitoring of lung function endpoints using home spirometry allowed accurate determination of the time course of improvements with UMEC. Both doses of UMEC demonstrated early benefits on home trough FEV_1_ by Week 1 that were sustained over the 24-week treatment period. This finding was supported by similar early benefits of UMEC on home PEF.

The magnitude of effect for clinic spirometry measurements obtained in this study were generally higher than home spirometry measurements. We speculate this difference may be due to conduct under supervision in a clinic setting. This difference may also be influenced by the time of day at which the measurements were performed, which was later for clinic than home measurements. However, as a treatment effect was observed for both doses of UMEC using both techniques, we conclude that while the use of home spirometry in individual patients may lack sensitivity, the increased frequency of home measurements and lack of systematic bias lends support to the accurate measurement of treatment effects when used in conjunction with more sensitive clinical measurements.

As with standard dose finding studies this study was not powered to directly compare the efficacy and safety of the two UMEC doses, but it can be used to inform appropriate doses for assessment in larger studies in the target population. It is notable that the magnitude of effect with UMEC 62.5 mcg versus placebo was generally numerically greater than UMEC 31.25 mcg versus placebo for all lung function endpoints. This was observed in the first 8 weeks of treatment with home trough FEV_1_ and at all time points up to and including Week 24, with UMEC 62.5 mcg also showing greater consistency of effect than UMEC 31.25 mcg when compared with placebo.

Numerical improvements in all PRO endpoints from baseline were observed in all treatment groups, including placebo, at Week 24. Although an MCID for E-RS total score has not been defined in asthma, both UMEC 31.25 mcg and 62.5 mcg showed a decrease of ≥2 points, while the placebo group did not. Notably, improvements in E-RS total scores were numerically greater with UMEC 62.5 mcg versus placebo than with UMEC 31.25 mcg versus placebo at all 4-weekly intervals, correlating with the observation that UMEC 62.5 mcg had consistent improvements for home trough FEV_1_ throughout the 24-week period, particularly over the latter half of the study. However, as this study was not adequately powered for statistical comparison of effects on PROs, a larger study in patients with asthma with a high burden of self-reported symptoms may be needed to more fully evaluate the effects of UMEC in patients with asthma.

Although this was not an exacerbation study, the annual rate and risk of experiencing an exacerbation was numerically lower with FF/UMEC versus FF alone, which supports previous findings showing that LAMAs can improve exacerbation rates and the time to first exacerbation in asthma [8, 10]. These results are also consistent with and of a similar magnitude to those observed with FF/VI versus FF alone, where the addition of a LABA to ICS reduced the rate of exacerbations [12, 31]. It is of interest that UMEC-treated patients experienced a numerically higher proportion of moderate than severe exacerbations while placebo-treated patients had a slightly higher proportion of severe exacerbations. It could be speculated that UMEC acts by preventing moderate exacerbations from becoming severe; however, the numbers of both moderate and severe events were small. Further studies in larger populations of patients with more severe asthma will be needed to confirm this potential effect of UMEC.

Importantly, the safety profile of UMEC/FF was as expected for this class of medications and no new signals were identified. No UMEC-dose-related increase in AEs was observed, as there was a higher incidence of AEs with UMEC 31.25 mcg compared with UMEC 62.5 mcg.

The study had a number of strengths, including the 24-week duration and the robust study design which ensured it was well powered to detect lung function improvements with centrally-measured spirometry. Furthermore, pulmonary function and symptoms were monitored daily, with patients required to record home trough FEV_1_ and PEF twice daily (AM and PM) and to use an eDiary to record asthma symptoms and rescue medication use. The requirement of daily E-RS total scores allowed for the standardisation of symptom assessment on a daily basis. Additionally, all measures were captured in the home environment when asthma may be at its worst (e.g., in the early AM). There was limited patient withdrawal, and patient follow up continued in patients following treatment cessation prior to 24 weeks. Finally, sensitivity analyses confirmed that the results were robust.

Study limitations included the relatively low burden of asthma symptoms of the included patients at study entry, which, combined with a limited run-in period and an effective background treatment, made differences in PRO endpoints between treatment groups difficult to detect in a study of this size, despite improvements in excess of the MCID for all treatment groups. The large placebo responses observed with PRO endpoints may reflect the beneficial effect of adherence to ICS (FF 100 mcg), which was maintained for 24 weeks. Including a longer run-in period with patients receiving ICS may have provided a more stable baseline and, in conjunction with enrolling patients with poorly controlled asthma (i.e., ACQ >1.5), could have potentially reduced the placebo response. Indeed, data on treatment compliance prior to the study were not collected, therefore, the effect of improved treatment compliance while taking part in the study could not be assessed. While our results provide support for the use of the E-RS in asthma, the E-RS was developed for use in patients with COPD and its modification for use in an asthma population needs to be validated before its routine use is appropriate. Finally, these results are restricted to a population with moderate asthma, limiting their relevance to other populations. However, the effect of UMEC was investigated in a Phase III study including patients with more severe asthma, uncontrolled on ICS/LABA (CAPTAIN, clinicaltrials.gov ID: NCT02924688), which will broaden the understanding of UMEC treatment in the treatment of asthma.

## Conclusions

UMEC is a highly effective once-daily bronchodilator that leads to improved lung function and is well tolerated in patients with fully reversible, uncontrolled/partially-controlled moderate asthma who are receiving ICS. Overall, these data support a favourable benefit/risk profile for UMEC (31.25 mcg and 62.5 mcg) in this asthma population.

## Supplementary information

**Additional file 1.** Supplementary Materials. Supplementary Tables 1–6.

## Data Availability

The anonymised individual participant data and study documents can be requested for further research from www.clinicalstudydatarequest.com.

## Abbreviations

ACQ: Asthma Control Questionnaire
AE: Adverse event
AESI: Adverse event of special interest
AM: Morning
AQLQ: Asthma Quality of Life Questionnaire
BMD: Bone mineral density
BMI: Body mass index
CAPTAIN: Clinical study of Asthma Patients receiving Triple therapy through A single INhaler
CFB: Change from baseline
CI: Confidence interval
CNS: Central nervous system
COPD: Chronic obstructive pulmonary disease
CV: Cardiovascular
DPI: Dry powder inhaler
eCRF: Electronic Case Report Form
eDiary: Electronic diary
EXACT: EXAcerbations of Chronic pulmonary disease Tool
E-RS: Evaluating Respiratory Symptoms
FEV_1_: Forced expiratory volume in 1 s
FF: Fluticasone furoate
FVC: Forced vital capacity
GINA: Global Initiative for Asthma
ICS: Inhaled corticosteroid
ITT: Intent-to-treat
LABA: Long-acting β_2_-agonist
LAMA: Long-acting muscarinic antagonist
LRTI: Lower respiratory tract infection
LS: Least squares
LTRA: Leukotriene receptor antagonist
MCID: Minimal clinically important difference
MDI: Metered dose inhaler
MedDRA: Medical Dictionary for Regulatory Activities
MMRM: Mixed model repeated measures
PEF: Peak expiratory flow
PM: Evening
PRO: Patient-reported outcome
SABA: Short-acting β_2_-agonist
SAE: Serious adverse event
SD: Standard deviation
SE: Standard error
SGRQ: St George’s Respiratory Questionnaire
SMQ: Standardised MedDRA queries
UMEC: Umeclidinium
US: United States
V: Visit

## References

[1] GBD 2015 Chronic Respiratory Disease Collaborators (2017). Global, regional, and national deaths, prevalence, disability-adjusted life years, and years lived with disability for chronic obstructive pulmonary disease and asthma, 1990–2015: a systematic analysis for the Global Burden of Disease Study 2015. Lancet Respir.

[2] Global Initiative for Asthma. Global Strategy for Asthma Management and Prevention 2019. Available from: www.ginasthma.org. Accessed 6 Jan 2020.

[3] National Institutes of Health (NIH) NH, Lung and Blood Institute. Expert Panel Report 3: Guidelines for the Diagnosis and Management of Asthma 2007. Available from: http://www.nhlbi.nih.gov/health-pro/guidelines/current/asthma-guidelines. Accessed 8 Feb 2019.

[4] Woodruff PG, Boushey HA, Dolganov GM, Barker CS, Yang YH, Donnelly S (2007). Genome-wide profiling identifies epithelial cell genes associated with asthma and with treatment response to corticosteroids. Proc Natl Acad Sci U S A.

[5] Demoly P, Annunziata K, Gubba E, Adamek L (2012). Repeated cross-sectional survey of patient-reported asthma control in Europe in the past 5 years. Eur Respir Rev.

[6] Food and Drug Administration. Highlights of prescribing information for SPIRIVA RESPIMAT. Available from: https://docs.boehringer-ingelheim.com/Prescribing%20Information/PIs/Spiriva%20Respimat/spirivarespimat.pdf. Accessed 26 Feb 2019.

[7] Boehringer Ingelheim Limited. Spiriva Respimat 2.5 microgram, inhalation solution. Summary of Product Characteristics. Available from https://www.spiriva.com/global/sites/default/files/spiriva_respimat_spc_march2018.pdf. Accessed 2 June 2020.

[8] Kerstjens HA, Casale TB, Bleecker ER, Meltzer EO, Pizzichini E, Schmidt O (2015). Tiotropium or salmeterol as add-on therapy to inhaled corticosteroids for patients with moderate symptomatic asthma: two replicate, double-blind, placebo-controlled, parallel-group, active-comparator, randomised trials. Lancet Respir Med.

[9] Kerstjens HA, Disse B, Schroder-Babo W, Bantje TA, Gahlemann M, Sigmund R (2011). Tiotropium improves lung function in patients with severe uncontrolled asthma: a randomized controlled trial. J Allergy Clin Immunol.

[10] Kerstjens HAM, Engel M, Dahl R, Paggiaro P, Beck E, Vandewalker M (2012). Tiotropium in asthma poorly controlled with standard combination therapy. N Engl J Med.

[11] Peters SP, Kunselman SJ, Icitovic N, Moore WC, Pascual R, Ameredes BT (2010). Tiotropium bromide step-up therapy for adults with uncontrolled asthma. N Engl J Med.

[12] Rodrigo GJ, Plaza V (2016). Once-daily fluticasone furoate and vilanterol for adolescents and adults with symptomatic asthma: a systematic review with meta-analysis. Ann Allergy Asthma Immunol.

[13] Decramer M, Maltais F, Feldman G, Brooks J, Harris S, Mehta R (2013). Bronchodilation of umeclidinium, a new long-acting muscarinic antagonist, in COPD patients. Respir Physiol Neurobiol.

[14] Lee LA, Yang S, Kerwin E, Trivedi R, Edwards LD, Pascoe S (2015). The effect of fluticasone furoate/umeclidinium in adult patients with asthma: a randomized, dose-ranging study. Respir Med.

[15] Lee L, Kerwin E, Collison K, Nelsen L, Wu W, Yang S (2017). The effect of umeclidinium on lung function and symptoms in patients with fixed airflow obstruction and reversibility to salbutamol: a randomised, 3-phase study. Respir Med.

[16] Leidy NK, Murray LT, Monz BU, Nelsen L, Goldman M, Jones PW (2014). Measuring respiratory symptoms of COPD: performance of the EXACT- respiratory symptoms tool (E-RS) in three clinical trials. Respir Res.

[17] Leidy NK, Sexton CC, Jones PW, Notte SM, Monz BU, Nelsen L (2014). Measuring respiratory symptoms in clinical trials of COPD: reliability and validity of a daily diary. Thorax..

[18] Nelsen LM, Lee LA, Wu W, Lin X, Murray L, Pascoe SJ, Leidy NK. Reliability, validity and responsiveness of E-RS:COPD in patients with spirometric asthma-COPD overlap. Respir Res. 2019;20(1):107. 10.1186/s12931-019-1070-6 PMC6545030 31151458

[19] Jones PW (2005). St. George's respiratory questionnaire: MCID. COPD..

[20] Juniper EF, Guyatt GH, Willan A, Griffith LE (1994). Determining a minimal important change in a disease-specific quality of life questionnaire. J Clin Epidemiol.

[21] Juniper EF, Svensson K, Mork AC, Stahl E (2005). Measurement properties and interpretation of three shortened versions of the asthma control questionnaire. Respir Med.

[22] Reddel HK, Taylor DR, Bateman ED, Boulet LP, Boushey HA, Busse WW (2009). An official American Thoracic Society/European Respiratory Society statement: asthma control and exacerbations: standardizing endpoints for clinical asthma trials and clinical practice. Am J Respir Crit Care Med.

[23] Virchow JC, Backer V, de Blay F, Kuna P, Ljorring C, Prieto JL (2015). Defining moderate asthma exacerbations in clinical trials based on ATS/ERS joint statement. Respir Med.

[24] Jones PW (2002). Interpreting thresholds for a clinically significant change in health status in asthma and COPD. Eur Respir J.

[25] Bleecker ER, Lotvall J, O'Byrne PM, Woodcock A, Busse WW, Kerwin EM (2014). Fluticasone furoate-vilanterol 100-25 mcg compared with fluticasone furoate 100 mcg in asthma: a randomized trial. J Allergy Clin Immunol Pract.

[26] Donohue JF (2005). Minimal clinically important differences in COPD lung function. COPD..

[27] Belloli EA, Wang X, Murray S, Forrester G, Weyhing A, Lin J (2015). Longitudinal forced vital capacity monitoring as a prognostic adjunct after lung transplantation. Am J Respir Crit Care Med.

[28] Finkelstein SM, Lindgren BR, Robiner W, Lindquist R, Hertz M, Carlin BP (2013). A randomized controlled trial comparing health and quality of life of lung transplant recipients following nurse and computer-based triage utilizing home spirometry monitoring. Telemedicine J e-Health.

[29] Menezes MB, Teixeira AL, Terra Filho J, Vianna EO (2008). Inflammatory and functional effects of increasing asthma treatment with formoterol or double dose budesonide. Respir Med.

[30] Russell AM, Adamali H, Molyneaux PL, Lukey PT, Marshall RP, Renzoni EA (2016). Daily home spirometry: an effective tool for detecting progression in idiopathic pulmonary fibrosis. Am J Respir Crit Care Med.

[31] Bateman ED, O'Byrne PM, Busse WW, Lotvall J, Bleecker ER, Andersen L (2014). Once-daily fluticasone furoate (FF)/vilanterol reduces risk of severe exacerbations in asthma versus FF alone. Thorax..

