# Supplementary material for: A phase IIb, randomised, parallel-group study: the efficacy, safety and tolerability of once-daily umeclidinium in patients with asthma receiving inhaled corticosteroids
Source: Respir Res. 2020 Jun 12;21:148. doi: 10.1186/s12931-020-01400-5 (PMC7291639; doi:10.1186/s12931-020-01400-5)
Supplement: Supplementary file 1 — Additional file 1. Supplementary Materials. Supplementary Tables 1–6. [file 12931_2020_1400_MOESM1_ESM.docx]

# Supplementary Materials

## Table S1. Summary of asthma maintenance therapy at study entry (ITT population).

| **Asthma maintenance therapy, n (%)** | **Placebo**  **(N=143)** | **UMEC 31.25 mcg**  **(N=139)** | **UMEC 62.5 mcg**  **(N=139)** | **Total**  **(N=421)** |
| --- | --- | --- | --- | --- |
| Monotherapy |  |  |  |  |
| ICS | 56 (39) | 45 (32) | 49 (35) | 150 (36) |
| Dual therapy | 81 (57) | 89 (64) | 87 (63) | 257 (61) |
| ICS/LABA | 80 (56) | 87 (63) | 84 (60) | 251 (60) |
| ICS/LTRA | 1 (<1) | 2 (1) | 3 (2) | 6 (1) |
| Triple therapy | 3 (2) | 4 (3) | 3 (2) | 10 (2) |
| ICS/LABA/LAMA | 1 (<1) **^†^** | 0 | 0 | 1 (<1) |
| ICS/LAMA/LTRA | 1 (<1) | 4 (3) | 3 (2) | 8 (2) |
| ICS/LABA/xanthines | 1 (<1) | 0 | 0 | 1 (<1) |
| No ICS-containing maintenance therapy | 3 (2)* | 1 (<1) | 0 | 4 (<1) |

*One patient was receiving ICS at study entry but the route of administration was incorrectly recorded as oral and the patient was counted under the no ICS-containing maintenance therapy.

**^†^**One patient was receiving ICS/LABA for ~1 year at study entry and UMEC for ~6 months. This treatment was halted 17 days prior to randomisation.

ICS, inhaled corticosteroid; ITT, intent-to-treat; LABA, long-acting β_2_-antagonist; LAMA, long-acting muscarinic antagonist; LTRA, leukotriene receptor antagonist; UMEC, umeclidinium

## Table S2. Baseline demographics by country (ITT population).

|  | **Poland (N=107)** | **Romania**  **(N=58)** | **United States (N=69)** | **Canada**  **(N=17)** | **Russian Federation (N=170)** | **Total  (N=421)** |
| --- | --- | --- | --- | --- | --- | --- |
| **Age, years** | 47.3 (14.04) | 48.9 (12.39) | 48.7 (16.07) | 43.9 (14.09) | 50.4 (15.12) | 48.8 (14.64) |
| **Female, n (%)** | 62 (58) | 42 (72) | 49 (71) | 12 (71) | 133 (78) | 298 (71) |
| **BMI, kg/m^2^** | 28.00 (5.38) | 27.47 (4.71) | 36.61 (10.61) | 33.38 (9.00) | 27.72 (5.72) | 29.44 (7.49) |
| **Treatment, n (%)**  **Placebo**  **UMEC 31.25 mcg**  **UMEC 62.5 mcg** | 34  35  38 | 21  20  17 | 25  22  22 | 5  7  5 | 58  55  57 | 143  139  139 |

Data are mean (SD) unless otherwise stated.

*Age is derived using the date of the pre-screening visit. Only year of birth is collected. Day and month of birth are imputed as 30 June.

BMI, body mass index; ITT, intent-to-treat; SD, standard deviation; UMEC, umeclidinium

## Table S3. Effect of UMEC versus placebo on home spirometry (ITT population).

| **Endpoint** (mean change from baseline) |  | **Time point** | **Placebo**  **(N=143)** | **UMEC 31.25 mcg**  **(N=139)** | **UMEC 62.5 mcg**  **(N=139)** |
| --- | --- | --- | --- | --- | --- |
| Home PM FEV_1_, L | n  LS mean change (SE)  Difference vs placebo (95% CI) | Weeks 1–24* | 140  -0.053 (0.025) | 133  0.022 (0.025)  0.076 (0.006, 0.145) | 134  0.029 (0.025)  0.082 (0.013, 0.152) |
| AM pre-dose PEF, L/min | n  LS mean change (SE)  Difference vs placebo (95% CI) | Weeks 1–24* | 142  -5.2 (3.74) | 137  17.4 (3.80)  22.6 (12.1, 33.1) | 138  18.0 (3.78)  23.2 (12.7, 33.7) |
| PM PEF, L/min | n  LS mean change (SE)  Difference vs placebo (95% CI) | Weeks 1–24* | 140  -9.7 (3.82) | 133  14.8 (3.91)  24.4 (13.6, 35.2) | 134  15.5 (3.89)  25.2 (14.4, 35.9) |

*Treatment effect averaged over all time points. These analyses were performed using an MMRM model with covariates of treatment, age, sex,

region, baseline value and 4-weekly period, and interaction terms for baseline value by period and treatment by period.

AM, morning; CI, confidence interval; FEV_1_, forced expiratory volume in 1 second; ITT, intent-to-treat; LS, least squares; MMRM, mixed model repeated measures; PEF, peak expiratory flow; PM, evening; SE, standard error; UMEC, umeclidinium.

## Table S4. Effect of UMEC versus placebo on rescue medication and PRO measures (ITT population).

| **Endpoint** (mean change from baseline) |  | **Time point** | **Placebo (N=143)** | **UMEC 31.25 mcg (N=139)** | **UMEC 62.5 mcg (N=139)** |
| --- | --- | --- | --- | --- | --- |
| Rescue medication use, puffs/day | n  LS mean change (SE)  Difference vs placebo (95% CI) | Weeks 1-24* | 139  -0.40 (0.08) | 131  -0.60 (0.08)  -0.20 (-0.4, 0.0) | 130  -0.50 (0.08)  -0.10 (-0.3, 0.1) |
| SGRQ total score | n  LS mean change (SE)  Difference vs placebo (95% CI) | Week 24 | 133  -9.97 (1.11) | 129  -11.47 (1.12)  -1.50 (-4.60, 1.60) | 131  -11.12 (1.11)  -1.15 (-4.24, 1.93) |
| SGRQ symptoms score | n  LS mean change (SE)  Difference vs placebo (95% CI) | Week 24 | 133  -17.06 (1.66) | 129  -21.10 (1.69)  -4.05 (-8.71, 0.62) | 131  -19.47 (1.68)  -2.42 (-7.06, 2.23) |
| SGRQ activity score | n  LS mean change (SE)  Difference vs placebo (95% CI) | Week 24 | 133  -9.33 (1.50) | 129  -13.18 (1.52)  -3.85 (-8.05, 0.35) | 131  -10.58 (1.51)  -1.25 (-5.45, 2.94) |
| SGRQ impact score | n  LS mean change (SE)  Difference vs placebo (95% CI) | Week 24 | 133  -8.08 (1.15) | 129  -7.85 (1.17)  0.23 (-3.01, 3.46) | 131  -8.82 (1.16)  -0.74 (-3.96, 2.48) |
| AQLQ total score | n  LS mean change (SE)  Difference vs placebo (95% CI) | Week 24 | 132  0.59 (0.07) | 128  0.61 (0.07)  0.02 (-0.16, 0.21) | 130  0.54 (0.07)  -0.05 (-0.24, 0.13) |
| ACQ-5 total score | n  LS mean change (SE)  Difference vs placebo (95% CI) | Week 24 | 135  -0.71 (0.06) | 130  -0.84 (0.06)  -0.14 (-0.31, 0.03) | 132  -0.77 (0.06)  -0.06 (-0.23, 0.10) |
| E-RS total score | n  LS mean change (SE)  Difference vs placebo (95% CI) | Weeks 1-24* | 140  -1.47 (0.33) | 134  -2.02 (0.34)  -0.55 (-1.48, 0.37) | 134  -2.29 (0.34)  -0.82 (-1.74, 0.10) |

*Treatment effect averaged over all time points. These analyses were performed using an MMRM model with covariates of treatment, age, sex, region, baseline value and 4-weekly period (rescue medication use or E-RS total score) or visit (other endpoints), and interaction terms for baseline value by period/visit and treatment by period/visit.

ACQ, Asthma Control Questionnaire; AQLQ, Asthma Quality of Life Questionnaire; CI, confidence interval; E-RS total score, Evaluating Respiratory Symptoms total score; ITT, intent-to-treat; LS, least squares; MMRM, mixed model repeated measures; PRO, patient-reported outcome; SE, standard error; SGRQ, St George’s Respiratory Questionnaire; UMEC, umeclidinium.

## Table S5. Responder analyses at Week 24 (ITT population).

|  |  | **Placebo**  **(N=143)** | **UMEC 31.25 mcg**  **(N=139)** | **UMEC 62.5 mcg**  **(N=139)** |
| --- | --- | --- | --- | --- |
| SGRQ total score | n  Responders, n (%)  Odds ratio vs placebo (95% CI) | 141  92 (65) | 137  95 (69)  1.25 (0.75, 2.09) | 138  90 (65)  1.00 (0.60, 1.66) |
| AQLQ total score | n  Responders, n (%)  Odds ratio vs placebo (95% CI) | 141  72 (51) | 136  72 (53)  1.15 (0.69, 1.91) | 138  73 (53)  1.17 (0.70, 1.94) |
| ACQ-5 total score | n  Responders, n (%)  Odds ratio vs placebo (95% CI) | 143  87 (61) | 139  89 (64)  1.17 (0.70, 1.95) | 139  85 (61)  1.11 (0.67, 1.84) |

Responder, defined by SGRQ total score decrease of ≥4 points from Baseline at Week 24, AQLQ total score increase of ≥0.5 from Baseline at Week 24, ACQ-5 score decrease of ≥0.5 from Baseline at Week 24. Responder analyses were conducted using a generalised linear model with a logit link function, covariates of treatment, age, sex, region, visit and baseline, and interaction terms of baseline by visit and treatment by visit.ACQ, Asthma Control Questionnaire; AQLQ, Asthma Quality of Life Questionnaire; CI, confidence interval; ITT, intent-to-treat; SGRQ, St George’s Respiratory Questionnaire; UMEC, umeclidinium.

## Table S6. Annualised rate of moderate/severe and severe asthma exacerbations (ITT population).

|  |  | **Placebo**  **(N=143)** | **UMEC 31.25 mcg**  **(N=139)** | **UMEC 62.5 mcg**  **(N=139)** |
| --- | --- | --- | --- | --- |
| Moderate/ severe | n  Annualised rate, mean (95% CI)  Rate ratio vs placebo (95% CI)  Reduction, % (95% CI) | 143  0.31 (0.19, 0.50) | 139  0.23 (0.13, 0.40)  0.74 (0.38, 1.45)  25.9 (-45.2, 62.2) | 139  0.24 (0.14, 0.41)  0.78 (0.40, 1.55)  21.7 (-54.5, 60.4) |
| Severe | n  Annualised rate, mean (95% CI)  Rate ratio vs placebo (95% CI)  Reduction, % (95% CI) | 143  0.10 (0.04, 0.22) | 139  0.03 (0.01, 0.10)  0.33 (0.12, 0.90)  67.3 (10.3, 88.1) | 139  0.05 (0.02, 0.13)  0.49 (0.19, 1.27)  50.7 (-26.8, 80.8) |

Analysis of exacerbation rate was performed using a negative binomial model with covariates of treatment, age, sex, region, severe asthma exacerbation in the previous year (0, ≥1) and with logarithm of time (year) on-study as an offset variable.

CI, confidence interval; ITT, intent-to-treat; UMEC, umeclidinium.
